# Study on the Dynamic Fracture Properties of Defective Basalt Fiber Concrete Materials Under a Freeze–Thaw Environment

**DOI:** 10.3390/ma17246275

**Published:** 2024-12-22

**Authors:** Guangzhao Pei, Dingjun Xiao, Miaomiao Zhang, Jiajie Jiang, Jiping Xie, Xiongzi Li, Junbo Guo

**Affiliations:** Department of Mining Engineering, School of Environment and Resources, Southwest University of Science and Technology, Mianyang 621010, China; pgz@mails.swust.edu.cn (G.P.); miaomiaozhang@mails.swust.edu.cn (M.Z.); j18882364841@mails.swust.edu.cn (J.J.); 17341934312@163.com (J.X.); bh13368301253@163.com (X.L.); dashiguo@126.com (J.G.)

**Keywords:** freeze–thaw cycles, basalt fiber, concrete, dynamic fracture toughness, crack propagation rate, crack resistance

## Abstract

This study examines the crack resistance of basalt-fiber-reinforced concrete (BFRC) materials subjected to freeze–thaw cycles (FTCs). We utilized a φ50 mm Split Hopkinson Pressure Bar (SHPB) apparatus alongside numerical simulations to carry out impact compression tests at a velocity of 5 m/s on BFRC specimens that experienced 0, 10, 20, and 30 FTCs. Additionally, we investigated the effects of basalt fiber (BF) orientation position and length on stress intensity factors. The results reveal that with an increasing number of FTCs, the dynamic crack propagation speed of BFRC with a prefabricated crack inclined at 0° rises from 311.84 m/s to 449.92 m/s, while its pure I fracture toughness decreases from 0.6266 MPa·m^0.5^ to 0.4902 MPa·m^0.5^. For BFRC specimens with a prefabricated crack inclination of 15°, the dynamic crack propagation speed increases from 305.81 m/s to 490.02 m/s, accompanied by a reduction in mode I fracture toughness from 0.3901 MPa·m^0.5^ to 0.2867 MPa·m^0.5^ and mode II fracture toughness from 0.6266 MPa·m^0.5^ to 0.4902 MPa·m^0.5^. In the case of a prefabricated crack inclination of 28.89°, the dynamic crack propagation speed rises from 436.10 m/s to 494.28 m/s, while its pure mode II fracture toughness decreases from 1.1427 MPa·m^0.5^ to 0.7797 MPa·m^0.5^. Numerical simulations indicate that fibers positioned ahead of the crack tip—especially those that are longer, located closer to the crack tip, and oriented more perpendicularly—significantly reduce the mode I stress intensity factor. However, these fibers have a minimal impact on reducing the mode II stress intensity factor. The study qualitatively and quantitatively analyzes the crack resistance of basalt-fiber-reinforced concrete in relation to freeze–thaw cycles and the fibers ahead of the crack tip, offering insights into the fiber reinforcement effects within the concrete matrix.

## 1. Introduction

Currently, the use of concrete is increasing in various engineering fields such as bridge, underground, protective, and marine engineering. These structures often face environments with low-frequency vibrations or low-speed impact loads, such as in subway segments, immersed tunnels, and underground storage facilities. Enhancing the impact resistance and crack resistance of concrete in such harsh conditions is crucial for expanding its application scope [1,2]. Randomly distributed fibers in fiber-reinforced concrete can significantly improve its mechanical properties [3]. Basalt fiber (BF), an inorganic fiber derived from basalt, is an environmentally friendly option [4]. Compared to plain concrete, basalt-fiber-reinforced concrete (BFRC) exhibits superior mechanical properties, including higher tensile strength [5], flexural strength [6], toughness [7], and impact resistance [8].

To enhance the frost resistance of concrete, numerous studies have been conducted, yielding significant findings. Li et al. [9] investigated the mass, microstructure, and porosity changes in basalt-fiber-reinforced concrete (BFRC) through freeze–thaw cycle (FTC) tests. Their results demonstrated that basalt fiber (BF) can refine the internal pore structure of concrete, leading to increased material density and significantly improved frost resistance. Further studies by Li et al. [10], Sahin et al. [11], and Kasim et al. [12] reported that, under identical FTC conditions, the compressive and flexural strengths of BFRC improved with increased BF content. Additionally, Gao et al. [13,14] examined the static and dynamic properties of basalt-fiber-reinforced cement soil and cemented clay after FTCs using static splitting tensile tests and Split Hopkinson Pressure Bar tests. Their findings indicated that BF significantly enhanced the static and dynamic tensile strengths of the mixtures post-FTCs. Zhao et al. [15,16,17] employed the digital image correlation (DIC) method to study the mechanical properties of BFRC after FTCs, revealing that BF improved the impact resistance of concrete and mitigated bending damage. However, existing research rarely addresses the influence of fiber application methods on the dynamic mechanical properties of freeze–thaw-damaged concrete. Therefore, exploring the effects of freeze–thaw actions and fiber application methods on the dynamic fracture performance of basalt fiber concrete is crucial for ensuring the safe use of concrete structures in cold and seasonally frozen regions.

In recent years, numerous scholars [18,19,20,21,22] have conducted in-depth studies on the fracture performance of fiber-reinforced concrete. The findings indicate that fibers enhance the concrete’s viscosity, restrict crack propagation in various directions, and effectively reduce crack width and number, thereby significantly improving the early crack resistance of concrete. Additionally, ductile fibers enhance the fracture performance of semi-rigid concrete matrices, reduce early crack formation, and impart superior toughness to fiber-reinforced concrete. Recent studies by Pirmohammad et al. [23,24,25,26,27] and Aliha et al. [28,29,30] have demonstrated that using different types of fibers and nanomaterials can improve the fracture resistance of asphalt concrete. Research [31,32,33] shows that uniformly distributed fibers significantly enhance the fracture performance of fiber-reinforced concrete, while fibers with smaller aspect ratios increase its fracture toughness and equivalent flexural strength.

In summary, basalt fiber concrete demonstrates enhanced frost resistance and crack resistance compared to plain concrete, with the distribution and orientation of the fibers playing a significant role in its fracture performance. Despite these benefits, research in this area remains limited. And basalt fiber-reinforced concrete contains a lot of microcracks, and the existence of microcracks will affect its crushing size during dynamic loading, which will lead to the instability of concrete engineering. The dynamic fracture toughness of concrete materials is an important parameter to evaluate the ability of concrete materials to resist dynamic crack initiation and propagation. This study aims to investigate the dynamic fracture mechanics of defective basalt fiber concrete under FTC conditions. Specimens were subjected to various FTCs using a rapid freeze–thaw method, and impact compression tests were conducted at a consistent loading rate utilizing a Split Hopkinson Pressure Bar (SHPB) apparatus. This study analyzes the impact of FTCs on the fracture mechanics of defective basalt fiber concrete. Additionally, ABAQUS 2020 numerical simulation software was employed to evaluate the effects of basalt fiber length, angle, and position on the stress intensity factor at the crack tip. The findings provide a valuable foundation for the construction and safety of concrete structures in cold, seasonally frozen regions. This study examines the relationship between FTCs, fibers ahead of crack tips, and the crack resistance of BFRC under dynamic impact loads. The findings can inform the construction and safety of concrete structures in cold, seasonally frozen regions.

## 2. Sample Preparation and Test Scheme

### 2.1. Preparation of Test Piece

This study involves the preparation of BFRC using water, cement, fine aggregate, coarse aggregate, and basalt fibers. The cement used was P.O 42.5 ordinary Portland cement. The fine aggregate was natural river sand with a fineness modulus of 2.5, and the coarse aggregate consisted of natural pebbles with a particle size range of 4–6 mm. Specific parameters of the BF are detailed in Table 1. The concrete grade designed for this experiment was C30. BFRC specimens were produced by incorporating chopped BFs into the concrete mix. Based on extensive research [34,35,36], this study examined basalt fiber concrete specimens with a volume fraction of 0.15%. This proportion is considered optimal as it enhances concrete strength without compromising workability or increasing material costs. Table 2 presents the relative weights of each component required for producing basalt fiber concrete.

### 2.2. Fracture Characteristic Test Scheme

**Figure 1:**
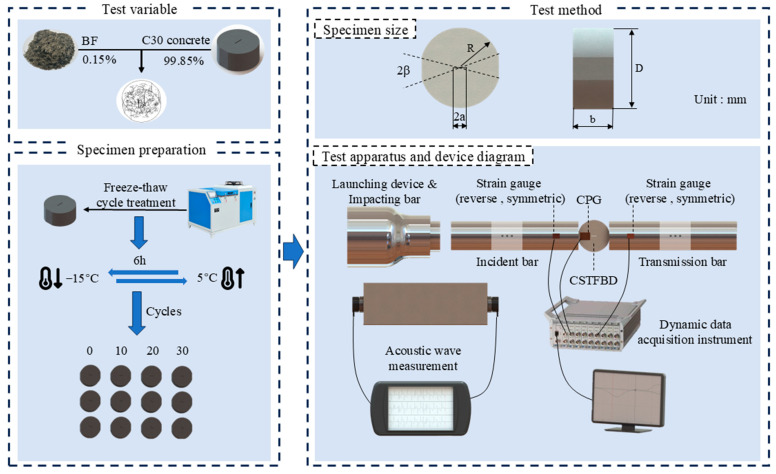
Test setup and flow chart.

The experimental procedure is outlined in Figure 1. The left side of the image illustrates the composition and freeze–thaw treatment of the samples, while the right side displays the appearance of the samples and the methods used for testing and inspection. This study employed a centrally cracked Brazilian disk specimen with a relative crack length of 2a/D = 0.2. To meet research requirements, pure mode I, pure mode II, and mixed mode I/II fracture tests were conducted. Based on relevant literature, the critical loading angle formula for pure mode II cracks is provided in Equation (1). Consequently, the loading angles selected for this experiment were 0° for pure mode I and 28.89° for pure mode II. A loading angle of 15° was chosen for the mixed mode I/II test. The cyclic conditions were established with a freezing temperature of −15 °C and a melting temperature of 5 °C, with the FTC lasting 6 h. Fracture characteristics were evaluated after 0, 10, 20, and 30 FTCs for the three specified angles.

(1) $$\begin{array}{c} \theta_{\mathrm{II}}=30.982-6.6667\alpha -19.048\alpha^{2}\\ \left(0.1<\alpha <0.9\right) \end{array}$$

The dynamic fracture characteristics were tested using an SHPB system with a bullet impact speed of 5 m/s. The experiment employed the DH5960 ultra-dynamic signal acquisition system and the Crack Propagation Gauge (CPG) to measure the crack initiation time and crack propagation speed of basalt fiber concrete subjected to varying FTCs. Additionally, a batch of standard prism specimens, measuring 100 mm × 100 mm × 400 mm, was cast alongside the BFRC specimens. The RSM-SY5 ultrasonic tester was then used to measure the transverse and longitudinal wave velocities of the BFRC after different FTCs. The typical longitudinal wave (C_P_) and transverse wave (C_S_) of basalt fiber concrete were measured. Using elastic wave theory [37,38], the Poisson’s ratio μ and elastic modulus of the material can be calculated, providing essential dynamic mechanical parameters for this numerical simulation [39,40,41].

## 3. Impact of FTCs on the Crack Resistance of BFRC

### 3.1. Impact of FTCs on the Crack Propagation Rate in BFRC and Microstructural Analysis

**Figure 2:**
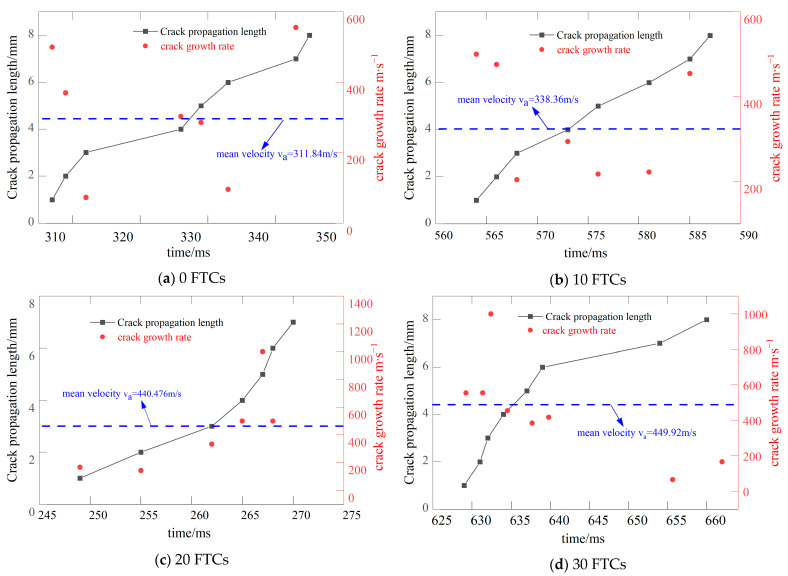
The crack tip position and crack propagation speed of the specimen when the inclination angle of the precast crack is 0.

Figure 2 illustrates the crack tip position and crack propagation speed of BFRC with a prefabricated crack inclination of 0° under varying FTCs. Regardless of the number of FTCs, the CPG consistently captures numerous voltage signals, allowing for the calculation of multiple instantaneous speeds. This results in a more accurate average speed calculation. This accuracy is attributed to the pure I fracture of the central straight crack platform in the Brazilian disc specimen when the prefabricated crack inclination is 0°, enabling the precise prediction of the crack propagation path. Additionally, it is observed that the average crack propagation speed increases progressively with the number of FTCs.

**Figure 3:**
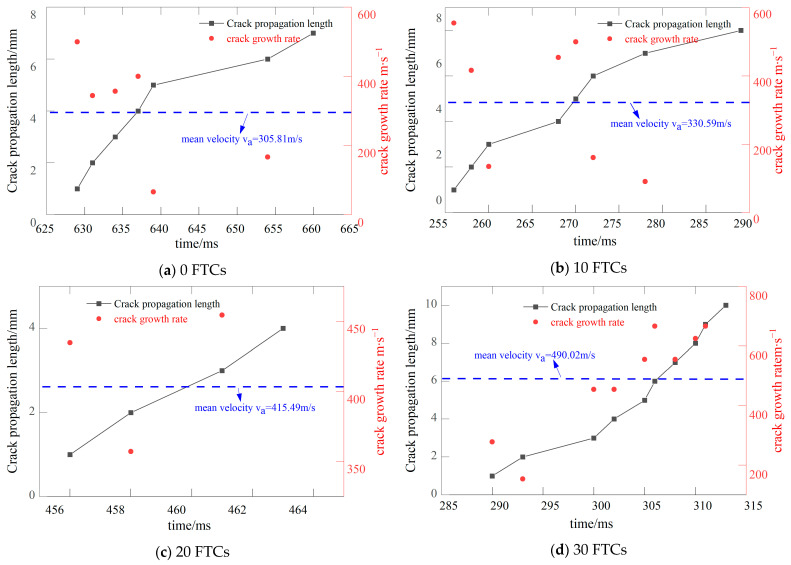
The crack tip position and crack propagation speed of the specimen when the precast crack inclination angle is 15.

Figure 3 illustrates the locations of crack tips and the rates of crack propagation in BFRC, featuring a prefabricated crack angle of 15° and subjected to various FTCs. At this angle, the specimens exhibit a mixed mode I/II fracture. The direction of crack propagation is more challenging to identify compared to a prefabricated crack angle of 0°, leading to a reduced number of signals captured by the CPG. After 20 FTCs, only four signals were recorded due to the material’s heterogeneity. The presence of aggregates within the specimen interferes with crack growth in the direction of the manually attached CPG, causing the crack to bypass the CPG once the initial four resistance wires fail. Nonetheless, it remains evident that the rate of crack propagation increases with the number of FTCs.

**Figure 4:**
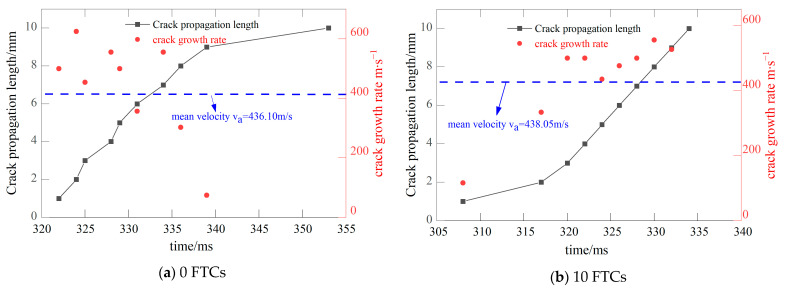

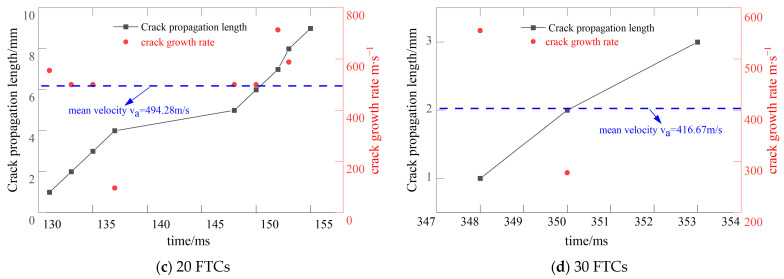
The crack tip position and crack propagation speed of the specimen when the precast crack inclination angle is 28.89.

Figure 4 illustrates the position of the crack tip and the crack propagation speed in BFRC with a prefabricated crack inclination of 28.89° across various FTCs. At this angle, the specimen exhibits a pure II fracture. It is observed that the average crack propagation speed generally increases with the number of FTCs. However, at 30 cycles, there is a notable decrease in propagation speed. This reduction can be attributed to the CPG capturing only three signals, which permits the calculation of just two instantaneous speeds, thus impacting the accuracy of the average speed calculation.

**Figure 5:**
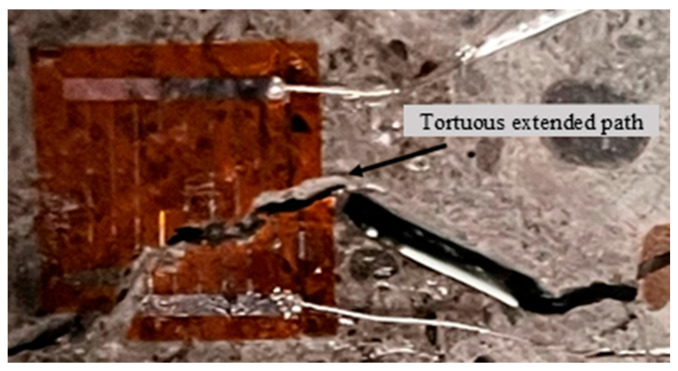
Crack propagation path.

Figure 5 illustrates the path of crack propagation in BFRC specimens, showing a meandering and tortuous trajectory. This phenomenon, along with related studies, indicates that crack propagation speed is consistently erratic [42,43]. Two primary factors can account for this phenomenon. First, the fundamental characteristics of brittle materials play a significant role. When subjected to impact loads, these materials exhibit rapid crack propagation. Upon reaching a critical threshold, this swift propagation results in fluctuations in speed, while the presence of parabolic grooves along the path further destabilizes the trajectory [44]. Secondly, the distinctive properties of concrete materials also contribute significantly. Concrete comprises randomly arranged aggregates, along with numerous microcracks and voids. As cracks propagate through the specimen, they often encounter microcracks, which accelerates their speed. Conversely, when cracks meet coarse aggregates, their speed decreases as they navigate through these regions. Additionally, when cracks reach the boundaries of coarse aggregates, the propagation path is altered. These factors collectively lead to the erratic speed and winding trajectory of crack propagation in materials such as concrete.

**Figure 6:**
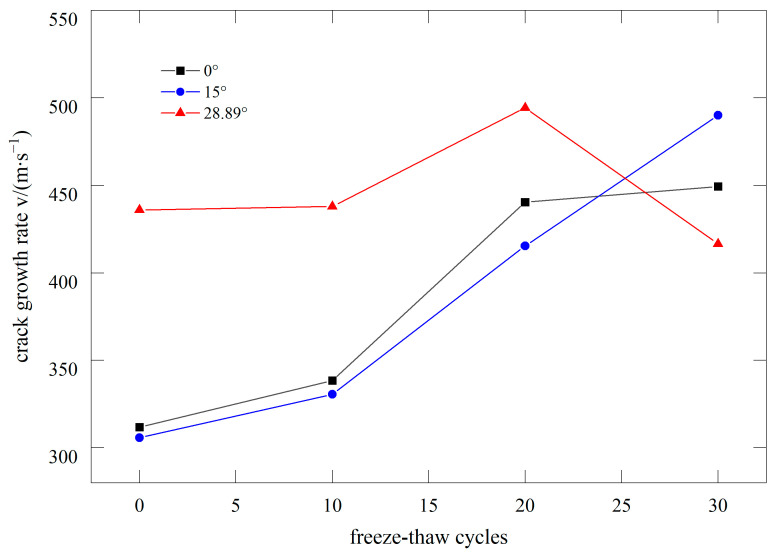
Relationship between dynamic crack growth rate and FTC.

**Figure 7:**
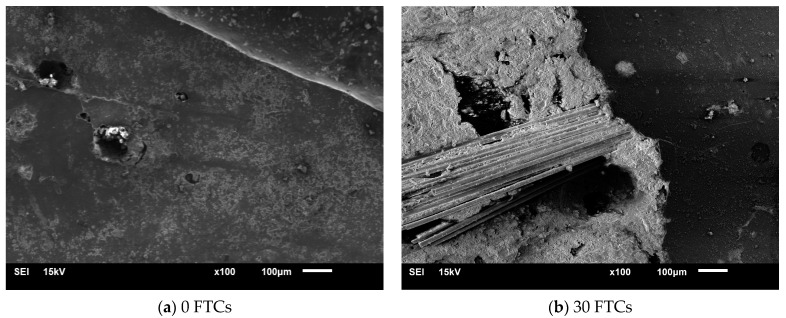
SEM images of basalt fiber concrete with different freeze–thaw times.

Figure 6 illustrates the relationship between the crack propagation speed of basalt fiber concrete under dynamic loading and the number of FTCs. Except for the data point with a prefabricated crack angle of 28.89° after 30 FTCs, which showed a decrease in average speed due to insufficient fracture data, the crack propagation speed generally increased with the number of FTCs. This is because BFRC experiences more severe degradation and develops numerous microcracks after undergoing additional FTCs. When the primary crack encounters these microcracks, the propagation speed accelerates. Using electron microscopy, we analyzed BFRC subjected to 0 and 30 FTCs. In the freeze–thaw medium, the concrete’s cement paste structure appeared loose, with fibers largely exposed. Energy spectrum analysis was conducted at two locations on the BFRC samples after 0 and 30 FTCs. The elemental content at these locations is shown in Table 3. After 0 cycles, the sample had the highest content of O and Ca, with lower levels of C and Si, indicating that SiO_2_ and C-S-H were the main components. After 30 cycles, O and Si were more concentrated, while Ca content decreased, suggesting that freeze–thaw damage disrupted the C-S-H structure, increasing SiO_2_ and reducing C-S-H content. This confirms that freeze–thaw action leads to an increase in microcracks (see Figure 7). Among the pre-existing crack angles, the dynamic crack propagation speed is highest at an angle of 28.89°, while the speeds at angles of 0° and 15° are similar. The higher energy release rate of mode II cracks results in a greater fracture velocity for these cracks.

### 3.2. Impact of FTCs on Fracture Toughness

**Figure 8:**
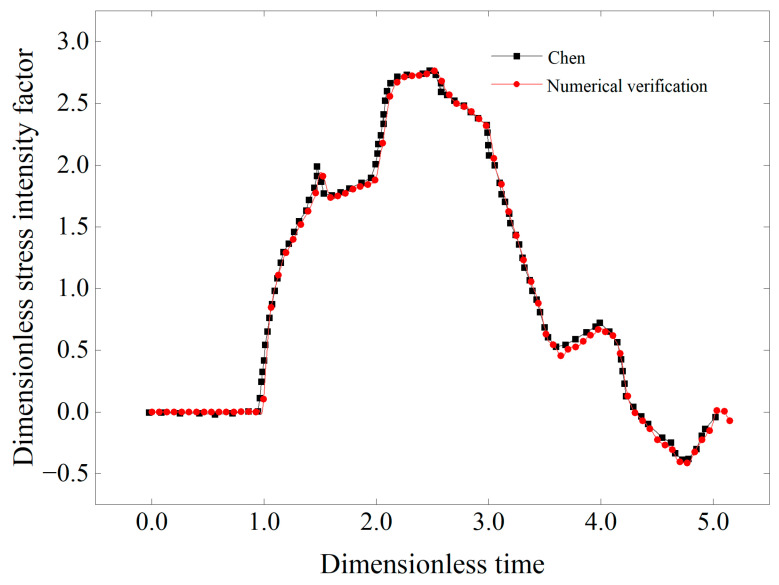
Numerical verification of Chen problem.

The fracture toughness of cracks at three different angles was evaluated under varying FTCs using an experimental–numerical analysis method. This approach involves importing dynamic load curves from experiments into a finite element model. By analyzing the variations in stress, strain, and displacement at the crack tip, a time history curve of the dynamic stress intensity factor can be created. The fracture toughness of the specimen is determined by identifying the stress intensity factor at the moment of crack initiation, as recorded by strain gauges positioned at the crack tip during the experimentation. For these calculations, the finite element analysis software ABAQUS was utilized. To validate the numerical results, the classic “Chen problem” [45] was first subjected to numerical verification, as illustrated in Figure 8. The “Chen problem” model consists of a two-dimensional plate measuring 40 mm in length and 20 mm in width, with a central crack of 4.8 mm. The plate has an elastic modulus of 200 GPa, a Poisson’s ratio of 0.3, and a density of 5000 kg/m^3^. A step load of 0.4 MPa is applied to both ends of the thin plate, resulting in a curve of the normalized stress intensity factor over time.

**Figure 9:**
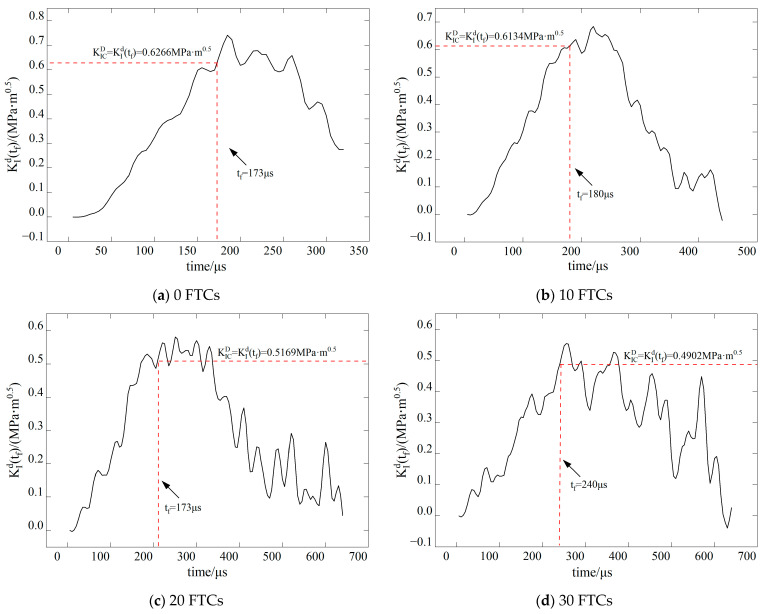
Time history curve of stress intensity factor *K*_I_ when the inclination angle of the precast crack is 0.

**Figure 10:**
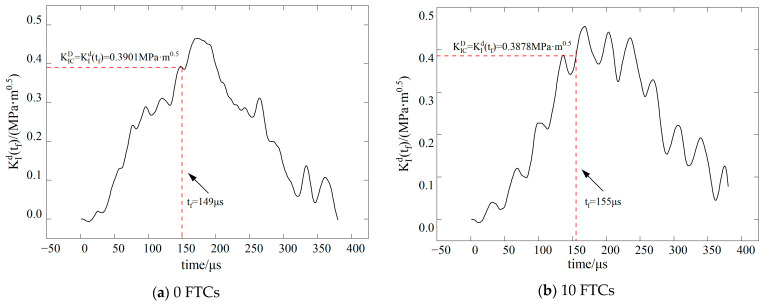

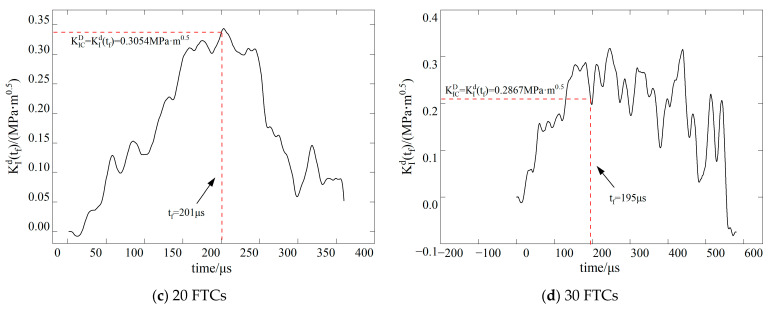
Time history curve of stress intensity factor *K*_I_ when the inclination angle of the precast crack is 15.

**Figure 11:**
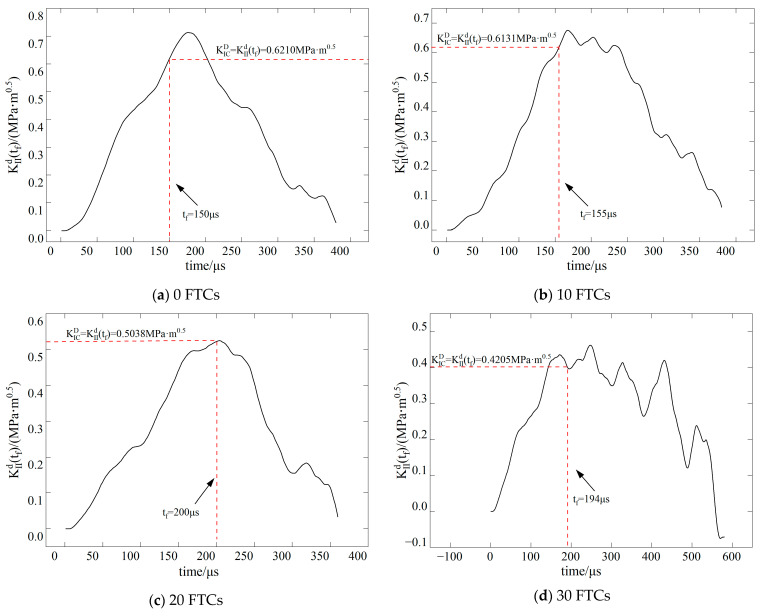
Time history curve of stress intensity factor *K*_II_ when the inclination angle of the precast crack is 15.

**Figure 12:**
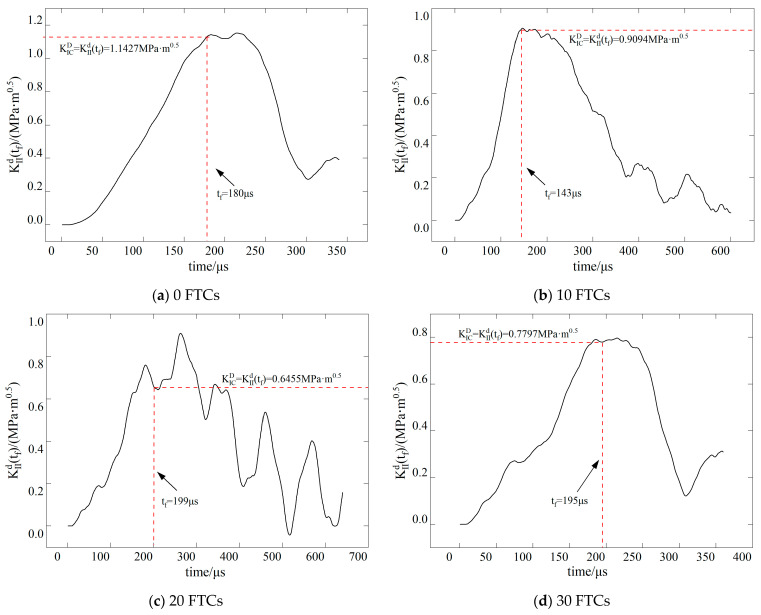
Time history curve of stress intensity factor *K*_II_ when the inclination angle of the precast crack is 28.89.

The dynamic stress intensity factor of BFRC under various conditions is shown in Figure 9, Figure 10, Figure 11 and Figure 12. In the figure, vertical dashed lines indicate the crack initiation moment ($t_{f}$) detected by CPG during the dynamic fracture test. By correlating this moment with the numerical simulation results, the stress intensity factor at this time represents the fracture toughness ($K_{IC}^{D}$) for this condition.

Table 4 presents the dynamic fracture toughness values $K_{I}^{d}$ and $K_{II}^{d}$ obtained from the experimental–numerical analysis method used in this dynamic fracture characteristics test.

**Figure 13:**
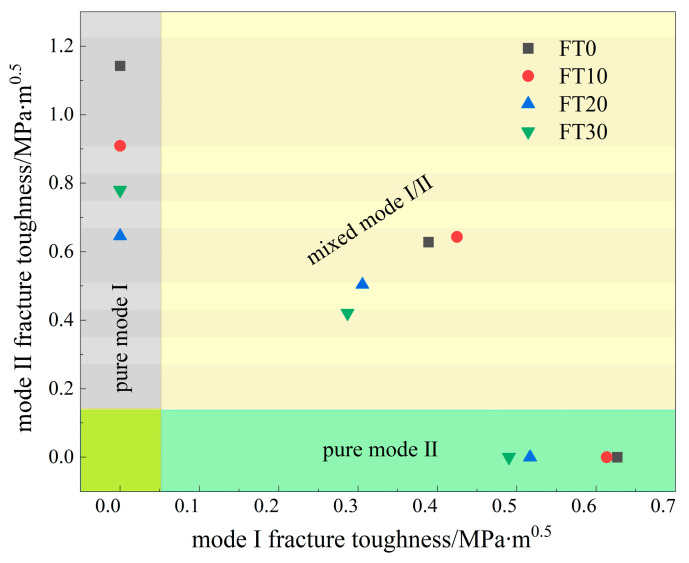
Fracture toughness of the Brazilian disc specimen with a straight crack platform in a dynamic fracture test center calculated by an experimental–numerical analysis method.

Figure 13 illustrates the dynamic fracture toughness calculated under various conditions using the experimental–numerical analysis method. The results indicate that the trend remains consistent across different conditions: fracture toughness decreases with an increasing number of FTCs. Specifically, when the prefabricated crack inclination is 0°, the fracture toughness $K_{I}^{d}$ of the specimen decreases by 2.10%, 15.73%, and 5.16% as the number of FTCs increases. For a prefabricated crack inclination of 28.89°, representing a pure II fracture, the fracture toughness $K_{II}^{d}$ decreases by 20.41% and 29.02% and then increases by 20.79% with additional freeze–thaw cycles. In the case of a 15° inclination, which corresponds to a mixed mode I/II fracture, both $K_{I}^{d}$ and $K_{II}^{d}$ decrease by 1.27%, 17.83%, and 16.53%, respectively, as the FTCs increase. The findings indicate that for pure I fracture, the most substantial reduction in fracture toughness occurs after 20 FTCs. Likewise, for mixed mode I/II fractures, the most notable decrease is observed at 20 cycles. In contrast, for pure mode II fractures, the greatest decline is also seen at 20 cycles; however, the fracture toughness shows a slight increase after 30 cycles compared to the 20-cycle mark.

## 4. Numerical Simulation of Dynamic Fracture Obstruction in Single Fibers

The stress intensity factor K depends on the load’s form and magnitude, the object’s shape, and the crack length. It characterizes the stress field intensity at the crack tip and serves as an indicator of whether the crack will become unstable. When the stress intensity factor at the crack tip reaches the fracture toughness, the crack begins to propagate. In practice, crack initiation and propagation are generally caused by excessive stress concentration at the crack tip. Excessive stress concentration primarily causes this, with the circumferential stress and shear stress at the crack tip having the most significant impact on crack propagation. In linear elastic fracture mechanics, the stress expressions in the local coordinate system are given by Equations (2) and (3).

(2) $$\sigma_{\theta \theta }=\frac{1}{2\sqrt{2\pi r}}\cos\frac{\theta }{2}\left[K_{I}\left(1+\cos\theta \right)-3K_{\mathrm{II}}\sin\theta \right]$$

(3) $$\tau_{r\theta }=\frac{1}{2\sqrt{2\pi r}}\cos\frac{\theta }{2}\left[K_{I}\sin\theta +K_{\mathrm{II}}\left(3\cos\theta -1\right)\right]$$

In the equations, $K_{1}$ and $K_{2}$ represent the stress intensity factors for mode I and mode II, respectively; θ is the angle of stress relative to the crack; $\sigma_{\theta \theta }$ denotes the circumferential stress; $\tau_{r\theta }$ represents the shear stress; and r is the radius from the crack tip. From Equation (2), it is evident that when θ is 0°, the crack is subjected only to circumferential tensile stress, and $K_{1}$ is directly proportional to $\sigma_{\theta \theta }$. From Equation (3), it is evident that when θ is 90°, the crack is influenced solely by shear stress, and $K_{2}$ is directly proportional to $\tau_{r\theta }$. Similarly, an increase in the stress intensity factor indicates more severe stress concentration at the crack tip, leading to a decrease in the material’s crack resistance.

**Figure 14:**
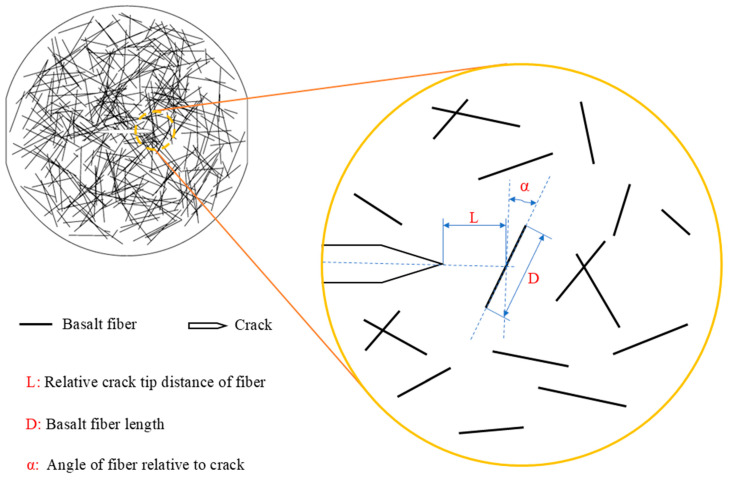
Fiber distribution before crack tip.

In Figure 14, the ABAQUS numerical simulation software was used to solve for the stress intensity factors of fibers located ahead of a crack tip under varying parameters L, D, and α. For the analysis, fibers of four different lengths (L = 5 mm, 10 mm, 15 mm, and 20 mm) were selected at a point 1 mm away from the crack tip in a direction perpendicular to the crack. Additionally, five fiber angles (α = 0°, 20°, 40°, 60°, and 80°) and five relative positions (D = 5 mm, 10 mm, 15 mm, 20 mm, and 30 mm) were considered. The dynamic load used for calculating the stress intensity factor curves corresponds to the dynamic load curve from the experiment with 0 FTCs. The concrete matrix is modeled using plane strain elements, while the fibers are represented by rod elements. An embedded operation integrates the fibers with the concrete, and the analysis is performed using a dynamic display method.

### 4.1. Impact of Fiber Length on Crack Resistance Performance of BFRC

**Figure 15:**
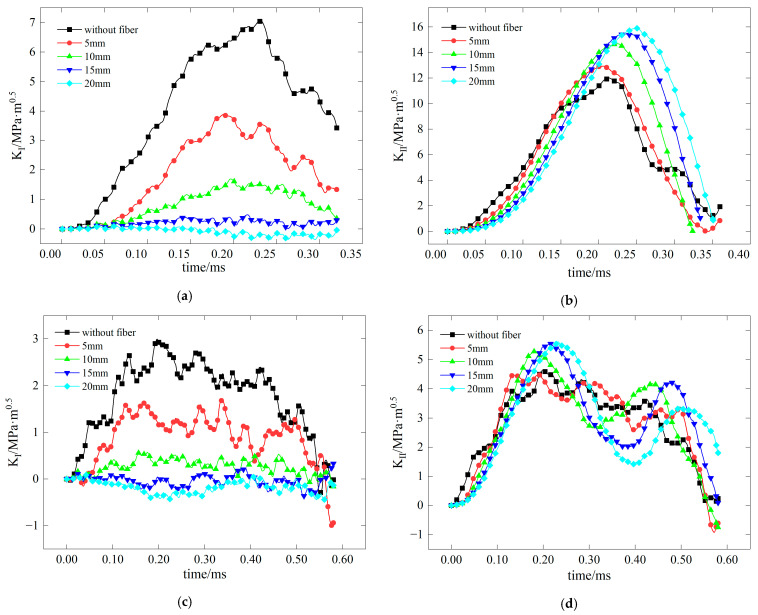
Stress intensity factors of concrete with different fiber lengths (**a**) *K*_I_ with a 0 inclination angle of precast crack; (**b**) *K*_II_ with a 28.89 inclination angle of precast crack; (**c**) *K*_I_ with a 15 inclination angle of precast crack; (**d**) *K*_II_ with a 15 inclination angle of precast crack.

The test examines the effect of varying fiber lengths on the stress intensity factors at crack tips, as illustrated in Figure 15, for pure I, pure II, and mixed mode I-II fractures. In the case of the pure I fracture, the stress intensity factor curve at the crack tip shows a decrease in amplitude with increasing fiber length. This indicates that longer fibers effectively reduce stress concentration and significantly improve the material’s resistance to mode I fractures. Conversely, for pure II fractures, the amplitude of the stress intensity factor curve increases with longer fibers, suggesting that these fibers weaken the material’s ability to resist mode II fractures, thereby exacerbating stress concentration. In mixed mode I–II fractures, longer fiber lengths notably enhance the material’s resistance to mode I fractures while diminishing its resistance to mode II fractures. When the fiber length reaches 15 mm, K_I_ is nearly 0, indicating the greatest improvement in mode I crack resistance.

**Figure 16:**
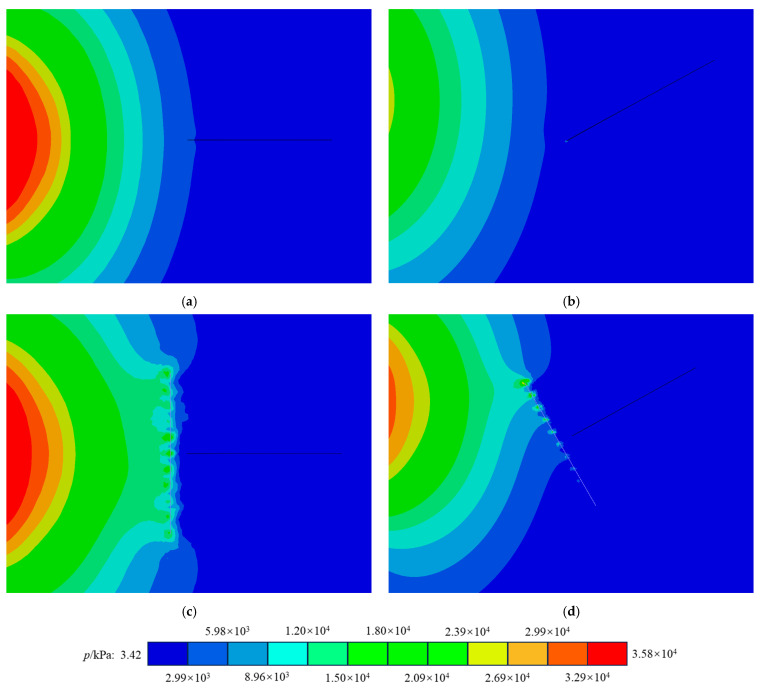
Force nephogram of fiber influence: (**a**) pure I stress nephogram; (**b**) pure II stress nephogram; (**c**) force nephogram of pure I fiber; (**d**) force nephogram of pure II fiber.

The primary reason BFs enhance the crack resistance of concrete is their higher elastic modulus compared to that of concrete itself. As shown in Figure 16, incorporating fibers can alleviate the rapid variations in the stress field relative to plain concrete. When the concrete matrix experiences impact, BFs help bear the load before the crack tip and absorb some of the energy, ultimately improving the concrete’s crack resistance. Longer fibers exhibit a tensile effect at an earlier stage, enabling them to better absorb external impacts. However, the inclusion of fibers and the increase in their length can negatively impact the mode II crack resistance of concrete. This occurs because the presence of fibers increases the stiffness disparity between the materials on either side of the crack, making mode II cracks more susceptible to sliding fractures. Consequently, in scenarios where enhanced mode I crack resistance is needed, incorporating longer basalt fibers can effectively reduce stress concentration at the crack tip, thus diminishing the likelihood of crack initiation in the concrete.

### 4.2. Impact of Fiber Orientation on Crack Resistance of BFRC

**Figure 17:**
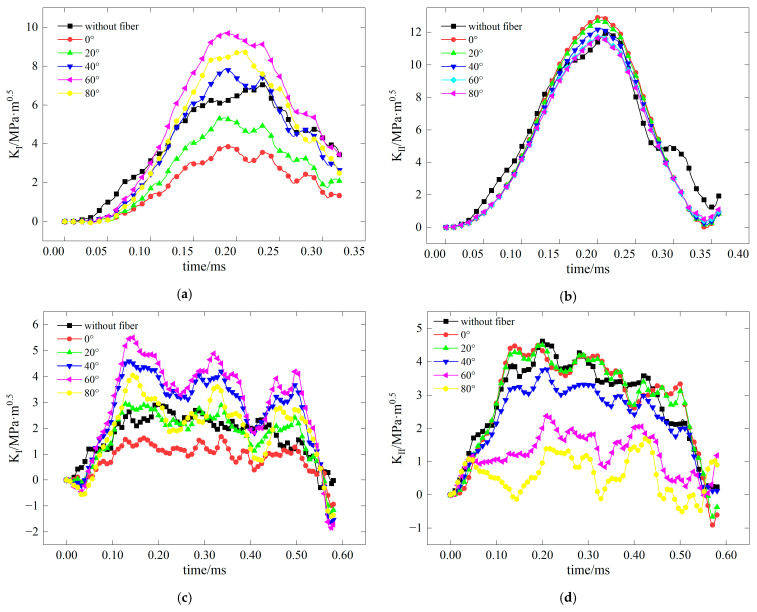
Stress intensity factors of concrete under different fiber angles (**a**) *K*_I_ with a 0 inclination angle of precast crack; (**b**) *K*_II_ with a 28.89 inclination angle of precast crack; (**c**) *K*_I_ with a 15 inclination angle of precast crack; (**d**) *K*_II_ with a 15 inclination angle of precast crack.

Figure 17 demonstrates the influence of fiber orientation on stress intensity factors. In pure I fracture, the stress intensity factor curve initially rises with increasing fiber angle before descending, reaching its peak at 60°. Below a fiber angle of 40°, the presence of fibers positively influences the mode I crack resistance of concrete. In contrast, in pure mode II fracture, the amplitude of the stress intensity factor curve exhibits a slight decline as the fiber angle increases. The differences in stress intensity factors between fiber-reinforced and plain concrete are minimal, although the inclusion of fibers may enhance the stress intensity factor. In mixed mode I/II fracture, an increase in fiber angle below 40° improves the mode I crack resistance but consistently undermines mode II crack resistance. When the fiber inclination angle is 0°, K_I_ decreases by approximately 50%, resulting in the greatest improvement in the mode I crack resistance.

**Figure 18:**
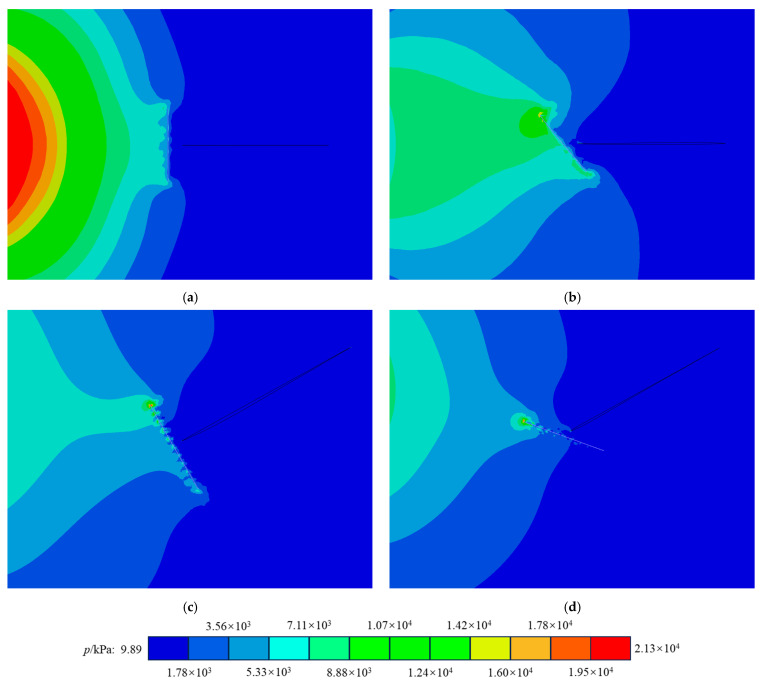
Force nephogram influenced by fiber angle: (**a**) pure I 0-degree fiber stress nephogram; (**b**) pure I 40-degree fiber stress nephogram; (**c**) pure II 0-degree fiber stress nephogram; (**d**) pure II 40-degree fiber stress nephogram.

Figure 18 illustrates that fibers oriented perpendicular to a crack are the most effective at inhibiting its propagation. When the fiber angle exceeds 40°, a significant asymmetry in the stress field near the crack tip occurs, resulting in an increase in the stress intensity factor. In contrast, as fibers become more parallel to the crack, the stress intensity factor decreases because the fibers do not effectively prevent crack opening and lack a strong asymmetric influence. Additionally, the introduction of fibers and variations in the fiber orientation have minimal effect on the mode II crack resistance of concrete and may even make mode II cracks more susceptible to fracturing.

### 4.3. Impact of Fiber Placement on Crack Resistance Performance of BFRC

**Figure 19:**
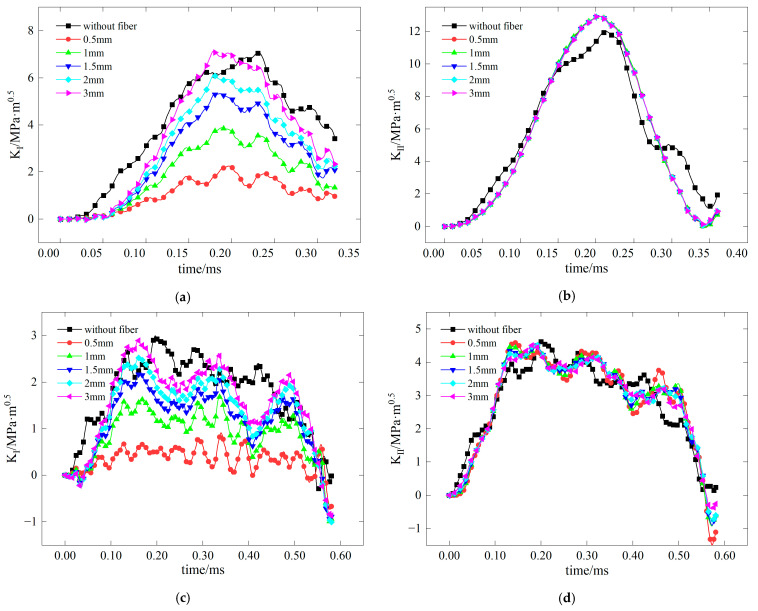
Stress intensity factors of concrete under different fiber positions: (**a**) *K*_I_ with a 0 inclination angle of the precast crack; (**b**) *K*_II_ with a 28.89 inclination angle of the precast crack; (**c**) *K*_I_ with a 15 inclination angle of the precast crack; (**d**) *K*_II_ with 15 inclination angle of the precast crack.

Figure 19 illustrates the effect of fiber position changes on stress intensity factors. In pure I fracture, the amplitude of the stress intensity factor curve decreases as the fiber distance increases, indicating that shorter distances between fibers and the crack tip significantly enhance the material’s mode I crack resistance. In pure II fracture, the amplitude of the stress intensity factor curve is not significantly affected, although there is a slight increase compared to plain concrete. This suggests that fibers at various distances have nearly the same weakening effect on the material’s mode II crack resistance. In mixed mode I/II fracture, the variation in the stress intensity factor curve follows the same pattern as in pure I and II fractures. When the fiber’s relative distance to the crack tip is 0.5 mm, it decreases the most; K_I_ is nearly 0, resulting in the greatest improvement in the mode I crack resistance performance.

**Figure 20:**
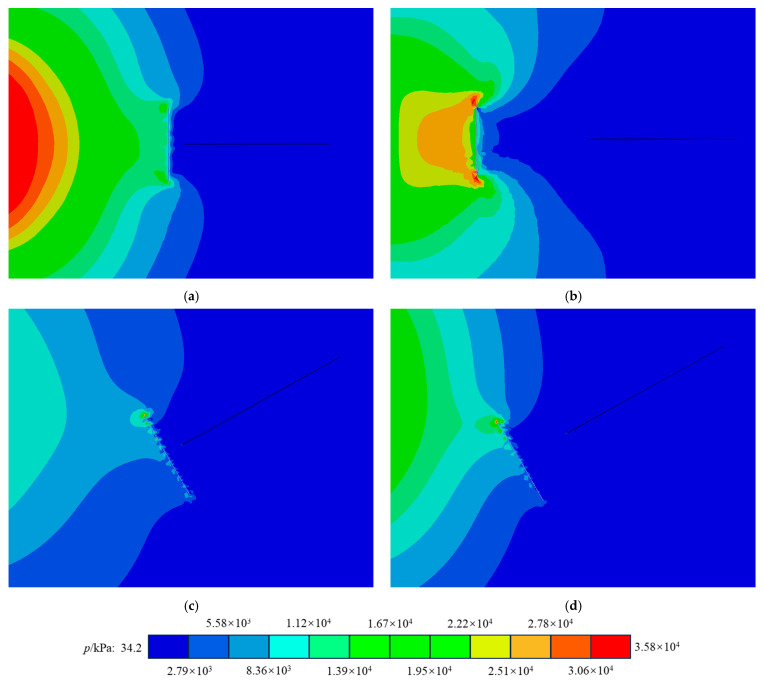
Fiber position affects the force nephogram: (**a**) force nephogram of pure I short-distance fiber; (**b**) force nephogram of pure I long-distance fiber; (**c**) force nephogram of pure II short-distance fiber; (**d**) force nephogram of pure II long-distance fiber.

Figure 20 demonstrates that fibers positioned nearest to the crack tip are most effective in inhibiting crack propagation. As these fibers move closer, they apply greater tensile force at the crack tip, thereby alleviating the concentrated stress present there. However, in the case of mode II fracture, the asymmetric effect of fibers at varying positions remains largely unchanged and may even exacerbate the stiffness disparity between the materials on either side of the crack. Consequently, this leads to increased stress differences on both sides of the crack tip, making mode II fractures more likely to occur.

## 5. Discussion

In general, the more pronounced the defects in concrete materials, the poorer their dynamic fracture performance tends to be [46]. This study investigates the dynamic fracture performance of BFRC materials under freeze–thaw conditions, specifically examining how the number of FTCs affects crack propagation speed and fracture toughness. At the microscopic level, scanning electron microscopy (SEM) analysis confirms that FTCs increase the micropores and fissures in BFRC. Furthermore, we hypothesize the presence of a single fiber within the concrete matrix to explore how the fiber length and orientation influence stress concentration at the crack tip. The numerical simulation results of the stress intensity factor, depicted in Figure 16, reveal that BFs significantly impact the propagation of stress waves within the concrete matrix. BFs play a positive role in mitigating the increase in stress concentration at the crack tip. These findings suggest that the enhancement of concrete’s crack resistance by BFs is primarily due to their tensile properties. Based on the findings of this study, it can be reasonably inferred that, under conditions where fibers are uniformly distributed, no stiffness differences appear on either side of cracks caused by the fibers in the contour plots (see Figure 18). The greater the load borne by the fibers at the crack tip, the more significant the improvement in the material’s resistance to mode I and mode II cracking. However, due to current research limitations, it is not possible to calculate the stress intensity factor for concrete containing a large number of uniformly distributed fibers.

Therefore, variations in fiber length and orientation are crucial for the crack resistance of concrete materials. This is particularly important in specific conditions, such as environments where concrete is frequently subjected to dynamic loads, leading to the formation of mode I cracks. During the pouring of concrete, incorporating longer basalt fibers and applying a magnetization process can align these fibers using a magnetic field. This alignment enhances the concrete’s resistance to mode I cracks in a specific direction [46]. Understanding the mechanism of dynamic crack resistance in BFRC can guide the efficient use and implementation of BFRC materials.

## 6. Conclusions

This study investigates the dynamic fracture characteristics of BFRC with defects under freeze–thaw conditions using CSTFBD specimens with varying prefabricated crack angles. It examines the impact of FTCs on crack propagation speed and employs an experimental–numerical analysis method to study the dynamic fracture toughness under different conditions. Additionally, ABAQUS software is used to explore the variations in stress intensity factors in CSTFBD specimens influenced by different fiber lengths and orientations, leading to the following conclusions:

(1) As the number of FTCs increases, the average crack propagation speed in defective BFRC also increases. Among these, the dynamic crack propagation speed is highest for prefabricated cracks with an inclination angle of 28.89°, while the speeds for angles of 0° and 15° are similar.
(2) The dynamic fracture toughness of defective BF concrete gradually decreases with an increasing number of FTCs. All three specimens exhibited the highest rate of decrease at 20 FTCs. Specifically, in pure I fracture, the fracture toughness decreased by up to 15.73%; in mode I-II mixed fractures, it decreased by up to 17.83%; and in the pure II fracture, the decrease reached 29.02%.
(3) The mode I crack resistance of BFRC improves when the fibers ahead of the crack tip are more perpendicular to the crack, longer, and closer to the crack tip. However, BFs do not enhance the mode II crack resistance of concrete.

## Figures and Tables

**Table 1:** Basic physical properties of basalt fiber.

Name	Length (mm)	Diameter (μm)	Density (kg·m^−3^)	Elastic Modulus (GPa)	Elongation (%)	Tensile Strength (MPa)
Basalt fiber	6	15	2600	95~115	2.4~3.1	3300~4500

**Table 2:** Mix proportion of basalt fiber concrete.

Water (kg·m^−3^)	Cement (kg·m^−3^)	River Sand (kg·m^−3^)	Broken Stone (kg·m^−3^)	Basalt Fiber (kg·m^−3^)
184	368	718	1122	6

**Table 3:** Element content ratio of samples before and after freeze–thaw cycle.

Freeze–thaw Times	Content/%	C-K	O-K	Na-K	Mg-K	Al-K	Si-K	Cl-K	K-K	Ca-K	Fe-K	Ti-K
0	weight	9.67	49.89	1.85	0.22	1.54	5.89	0.55	0.63	26.83	2.93	0
	atom	16.00	61.95	1.59	0.18	1.14	4.17	0.31	0.32	13.30	1.04	0
30	weight	11.41	50.14	2.45	2.19	5.00	15.91	0	0.86	8.29	3.24	0.51
	atom	17.83	58.80	2.00	1.69	3.47	10.63	0	0.41	3.88	1.09	0.20

**Table 4:** Fracture toughness calculated by the experimental–numerical analysis method.

Series	$K_{I}^{d}$/MPa·m^0.5^	$K_{II}^{d}$/MPa·m^0.5^
D-0-0	0.6266	0
D-10-0	0.6134	0
D-20-0	0.5169	0
D-30-0	0.4902	0
D-0-15	0.3901	0.6210
D-10-15	0.3878	0.6131
D-20-15	0.3054	0.5038
D-30-15	0.2867	0.4205
D-0-28.89	0	1.1427
D-10-28.89	0	0.9094
D-20-28.89	0	0.6455
D-30-28.89	0	0.7797

## Data Availability

The original contributions presented in this study are included in the article. Further inquiries can be directed to the corresponding author.
